# Identification of a Step-And-Brake Controller of a Human Based on Prediction of Capturability

**DOI:** 10.3389/frobt.2022.729593

**Published:** 2022-04-28

**Authors:** Miharu Kojima, Tomomichi Sugihara

**Affiliations:** ^1^ Department of Adaptive Machine Systems, Graduate School of Engineering, Osaka University, Suita, Japan; ^2^ OMRON Corporation, Tokyo, Japan

**Keywords:** biped locomotion, step-and-brake motion, human motor control, COM-ZMP model, system identification, capturability

## Abstract

An explicit mathematical form of a human’s step-and-brake controller is identified through motion measurement of the human subject. The controller was originally designed for biped robots based on the reduced-order dynamics and the model predictive control scheme with the terminal capturability condition, and is compatible with both stand-still and stepping motions. The minimal number of parameters facilitates the identification from measured trajectories of the center of mass and the zero-moment point of the human subject. In spite of the minimality, the result only suited the human’s behaviors well with slight modifications of the model by taking direction-dependency of the natural falling speed and the inertial torque about the center of mass into account. Furthermore, the parameters are successfully identified even from the first half of motion sequence, which means that the proposed method is available in designing on-the-fly systems to evaluate balancing abilities of humans and to assist balances of humans in walking.

## Introduction

Biped locomotion is one of the fundamental functions of humans. It is valuable to understand how skillful behaviors for locomotion are synthesized not only from a scientific but also a practical viewpoint as it suggests efficient methods for medical diagnoses, rehabilitations, assistive system designs, for example. While lots of knowledge about how human body parts contribute to walking has been accumulated particularly in the fields of physiology and clinical medicine, it is also necessary to know how those parts are coordinated into the whole-body motion in order to properly diagnose and effectively aid humans’ locomotion abilities. In this regard, it has been demanded to find a plausible mathematical model of the humans’ locomotion controller (Cavagna and Margaria, 1966; Yamashita et al., 1972; Alexander, 1976; McMahon, 1984; Ren et al., 2006; Schmitthenner and Martin, 2021), which is still challenging.

Many studies (Taga, 1995; Hase and Yamazaki, 1998; Xiang et al., 2011; Shachykov et al., 2019) to reproduce locomotive behaviors on a multi-link neuro-musculoskeletal system model in computer simulations have been made. When aiming to describe the humans’ control mechanism in a comprehensible manner rather than to simulate biological behaviors precisely, it is reasonable to focus on the reduced dynamics of the whole-body. The center of mass (COM) is a point into which effective inertia of the body is condensed, and hence, has been studied in many works (Whittle, 1997; Lee and Farley, 1998; Hof, 2008). It is also known (Mitobe et al., 2000) that the macroscopic dynamics of the overall anthropomorphic system can be represented based on the relationship between the COM and the center of pressure (COP), which is also called the zero-moment point (ZMP) (Vukobratović and Stepanenko, 1972) in the field of robotics.

Even if we only focus on the reduced dynamics, it is not easy to understand the mechanisms of the behavior to carry a foot from the current place to another since it is accompanied with a complex coordination of the COM and the ZMP. A dynamic loading and unloading on the both soles and a smooth transition of the pivot foot are required during the process; while a foot to step should be unloaded in order to lift off the ground, the same foot initially has to be loaded in order to accelerate the COM appropriately.

The goal of this work is to find a mathematical model of a controller of a human’s step-and-brake motion as the minimum motion unit of the above process. The authors (Sugihara et al., 2022) have learned that control techniques developed for biped robots potentially explain the humans’ control schemes because of the morphologic, and accordingly, dynamical similarity between humanoid robots and humans. Among a number of controllers proposed in the field so far, Sugihara et al.’s method (Sugihara and Yamamoto, 2017; Yamamoto and Sugihara, 2020) was picked up as the primary candidate of the model due to its minimal property in a sense that it is derived from the minimal conditions to stabilize a biped robot from the minimal number of parameters and state variables based on the terminal capturability (Pratt et al., 2006; Koolen et al., 2012). Parameter identifications of the model were conducted from measured motion trajectories of a human subject. Though the authors predicted at first that the model would be only a starting point and need a lot of improvements to fill in the gaps from the actual behavior of a human, the model surprisingly fitted it with slight modifications, which was a consideration of direction-dependent time-constant of natural falling and the inertial torque about the COM. A contribution of this work over the previous researches which focused on the capturability during the stance (Hof, 2008) and that predicted landing positions of feet (Aftab et al., 2012) is that a model of the feedback controller that reproduces the overall dynamic stepping motion process was identified in a mathematically explicit form in it. We also note that our result has been already utilized in another work (Yoshikawa, 2022).

An early version of this work was presented at the eighth IEEE RAS/EMBS International Conference on Biomedical Robotics and Biomechatronics (Kojima and Sugihara, 2020). A clear modification in this paper from the version is at the last part of Section 5. We newly confirmed that the control parameters could be estimated only from the first half movement before completing the action. It means that the model is available in designing on-the-fly systems, for example, to evaluate balancing abilities of humans and to assist balances of humans in walking.

## COM-ZMP Model for Human Dynamics

**FIGURE 1:**
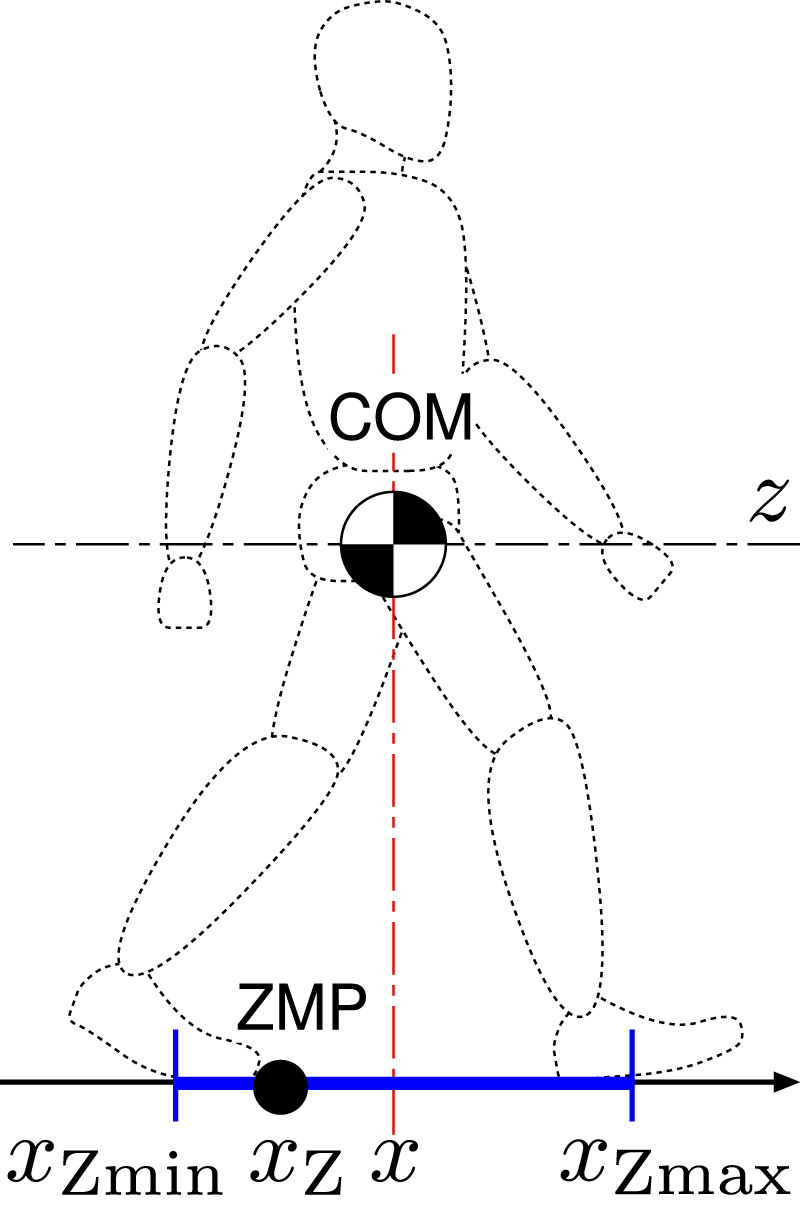
The COM-ZMP model for human dynamics in the sagittal plane. It is additionally assumed that the vertical movement of COM and the inertial torque about COM are negligibly small.

The COM-ZMP model (Mitobe et al., 2000) is often employed in the field of humanoid robotics as a reduced-order representation of dynamics of the robot. This is also available for the human dynamics analysis. Let us consider a human’s motion on the sagittal plane as illustrated in Figure 1. Suppose both the vertical movement of the COM and the inertial torque about the COM are negligibly small. The equation of motion of the COM is represented as

$$\ddot{x}=\zeta^{2}\left(x-x_{\text{Z}}\right)$$ (1)

$$\zeta \overset{def}{=}\sqrt{\frac{g}{z}}\;\text{:const.},$$ (2)

where *x* and *x*_Z_ are the longitudinal positions of the COM and the ZMP, respectively, *z* is the height of the COM with respect to the nominal ground, which is assumed to be constant, and *g* = 9.8 m/s^2^ is the acceleration due to the gravity. For the mathematical derivation, refer Sugihara and Morisawa (2020). The ZMP is naturally constrained within the supporting region due to the unilaterality of the contact forces as

$$x_{\text{Zmin}}\leq x_{\text{Z}}\leq x_{\text{Zmax}},$$ (3)

where *x*_Zmin_ and *x*_Zmax_ are the rear and front ends of the supporting region in *x*-axis, respectively. We also assume that the human can gain a sufficient friction force from the ground, so that its limitation is not taken into account.

The COM-ZMP model is utilized in several contexts such as motion planning (Kajita et al., 2003; Nagasaka et al., 2004; Harada et al., 2006; Sugihara and Nakamura, 2009) and control (Mitobe et al., 2000; Sugihara et al., 2002; Morimoto et al., 2008; Sugihara, 2009). The authors (Sugihara et al., 2022) found that the COM-ZMP regulator (Sugihara, 2009) fairly models a standing stabilization behavior of a human through a system identification technique. Although it is not directly related with the step-and-brake control, it provides some relevant knowledge about the study in this paper. Hence, we summarize it here in order to deepen the following discussions.

The COM-ZMP regulator is a controller to stabilize the COM of a humanoid in stance, in which the desired ZMP ^*d*^ *x*_Z_ is decided by a piecewise-linear feedback of the state of the COM as

xZd=xZmaxS1:x~Zd≥xZmaxx~ZdS2:xZmin<x~Zd<xZmaxxZminS3:x~Zd≤xZmin (4)

x~Zd=defxd+k1x−xd+k2x˙, (5)

where ^*d*^ *x* is the referential position of the COM and *k*_1_ and *k*_2_ are feedback gains. Note that this is equivalent with determining the desired net ground reaction force under the assumption that the COM keeps the constant height. Suppose the actual ZMP is manipulated so as to track the desired ZMP accurately, i.e., *x*_Z_ = ^*d*^ *x*_Z_. The feedback system becomes piecewise-affine as

x¨=ζ2x−ζ2xZmaxS1−ζ2k1−1x−xd−ζ2k2x˙S2ζ2x−ζ2xZminS3. (6)

When the system poles in (S2) are − *ζq*_1_ and − *ζq*_2_, the following equation holds:

$$k_{1}=q_{1}q_{2}+1,\;k_{2}=\frac{q_{1}+q_{2}}{\zeta }.$$ (7)

**FIGURE 2:**
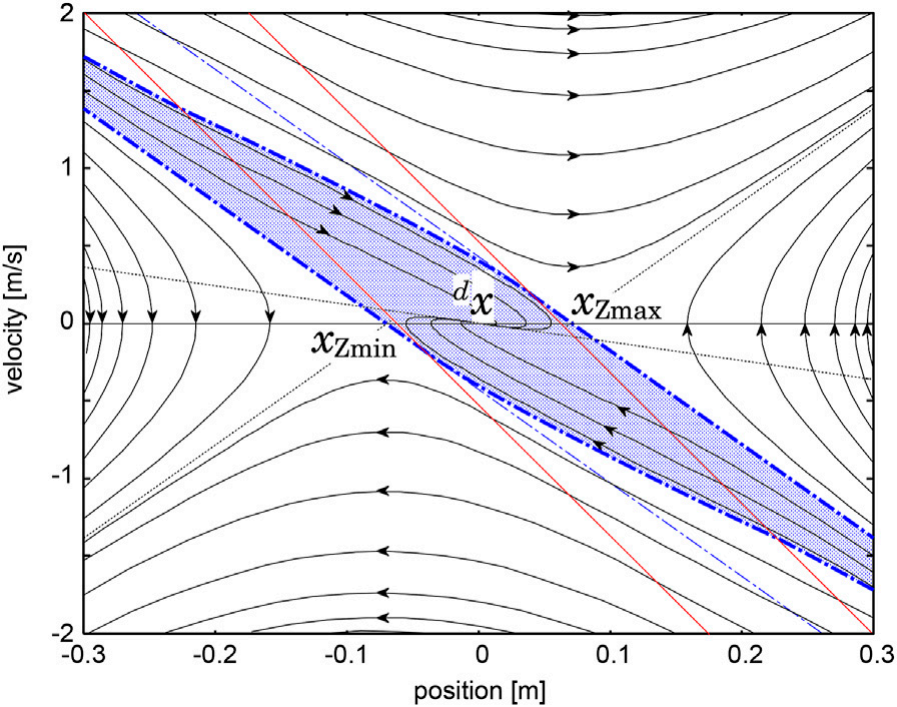
Theoretical phase portrait of the COM-ZMP regulator represented by Eqs 1, 3, 4 with $\zeta =\sqrt{9.8/0.27}≃6.0$ and (*q*_1_, *q*_1_) = (0.2, 0.6).

Figure 2 shows a typical example of a phase portrait of the system defined by Eq. 6 with $\zeta =\sqrt{9.8/0.27}≃6.0$ and (*q*_1_, *q*_2_) = (0.2, 0.6).

**FIGURE 3:**
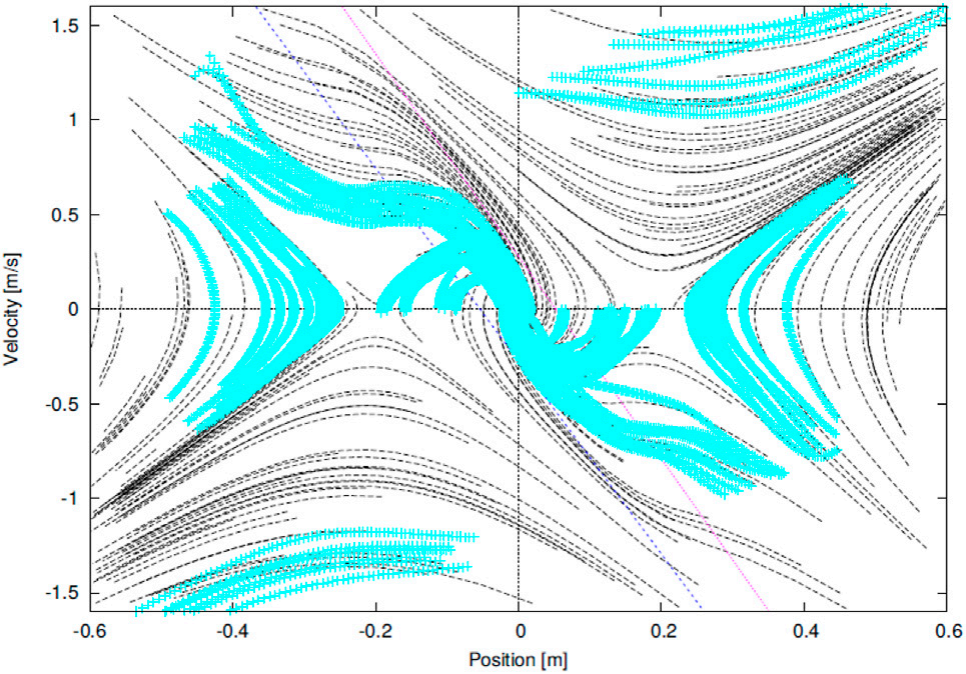
phase portrait of an identified COM-ZMP regulator from a human’s behavior in our previous work (Sugihara et al., 2022).

A human’s standing controller was identified from motion trajectories measured by an optical motion capture system under a hypothesis that the human’s behavior is modeled by Eq. 6. Figure 3 shows the result in the lateral plane, in which cyan points are samples of the measured trajectories and solid lines are trajectories of the identified dynamical system. Refer the original paper for the detail. The resemblance of Figures 2, 3 qualitatively supports the hypothesis. A quantitative analysis made in the original paper revealed a worth mentioning difference between them that the two asymptotic lines in (S1) and (S3) are not symmetric with respect to the axes, which means that the time-constant of natural falling motion depends on the direction. This characteristic is magnified in the longitudinal direction, and thus, will be reconsidered later.

## Measurement of Step-And-Brake Motions

**FIGURE 4:**
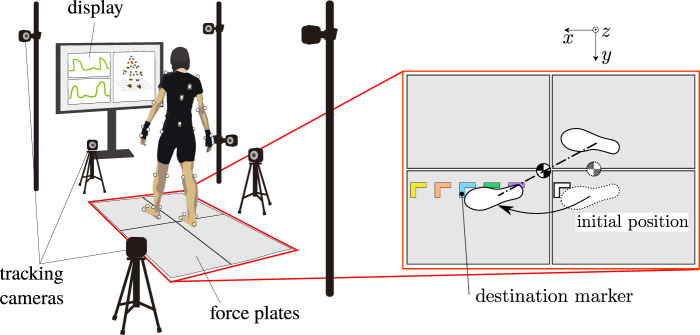
Set-up of the motion measurement system with 11 infrared cameras and 4 force plates. The foot-landing locations are specified by marker tapes put on the force plates.

**FIGURE 5:**
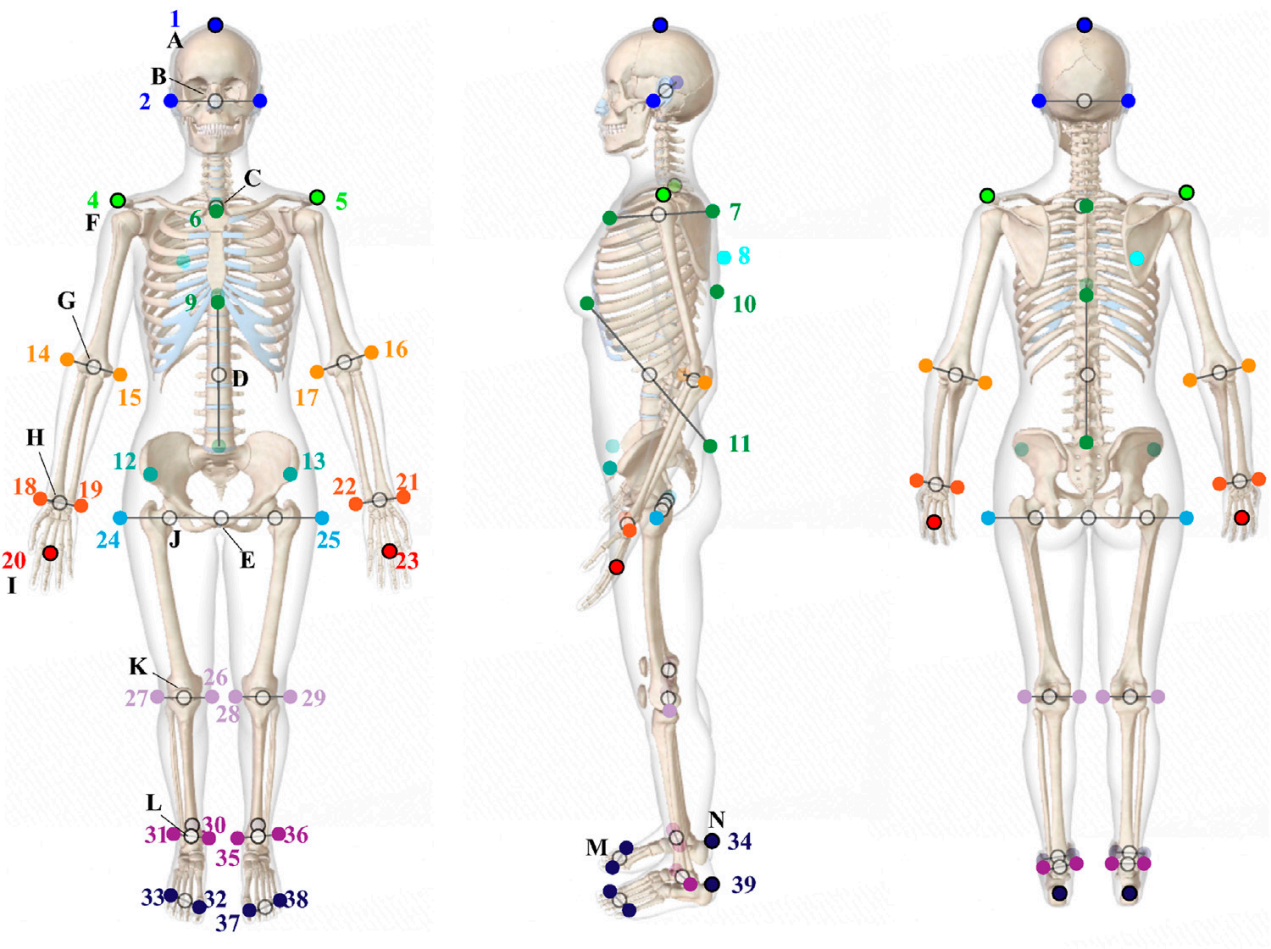
Definitions of 39 retroreflective markers attached on the subject’s body for the motion measurement.

Motion measurement experiments were conducted in order to observe movements of the COM, the ZMP and the feet during the step-and-brake motions. Figure 4 illustrates a set-up of the measurement system. 3D trajectories of 39 retroreflective markers attached on the subject’s body according to Figure 5 were measured at 240 Hz of the sampling rate by an optical motion capture system (VENUS3D; Nobby Tech. Ltd.) with 11 infrared cameras. At the same time, the reaction forces and torques from the ground were measured by force plates (TF-6090; Tech Gihan Inc.,). The forces exerted to each foot were separately measured by different plates. The trajectories of the ZMP were computed from the reaction forces and torques. The subject was a 23-year-old healthy female, who was 158 cm tall and weighed 48 kg. She was informed the objective and risk of the experiment and understood them in advance. In each trial, she initially took a standing posture, got her left-foot to step onto a location specified by tapes put on the force plates as depicted in the right side of Figure 4, and braked herself immediately after landing. The tapes were put in 25 ∼65 cm range from the initial position with 10 cm intervals. The stepping durations were controlled by a metronome at 100 bpm. The referential position of the COM to settle was not visually indicated, but the reaction forces on each foot were displayed in real-time in a monitor in front of the subject, which guided her to balance them and locate the COM at the middle of the both feet. Eight trajectories with respect to each designated location to step were collected. Hence, the total number of the trajectories was 40. The trajectories of the COM were estimated based on a standard mass-distribution model of Japanese adult females (Ae et al., 1992) that was scaled in accordance with the subject’s body proportion. Measurement noises were reduced by a second-order Butterworth filter with 5 Hz of cutoff frequency, which was tuned by trial and error. A history of velocity and acceleration of the COM were computed by the midpoint finite difference method.

**FIGURE 6:**
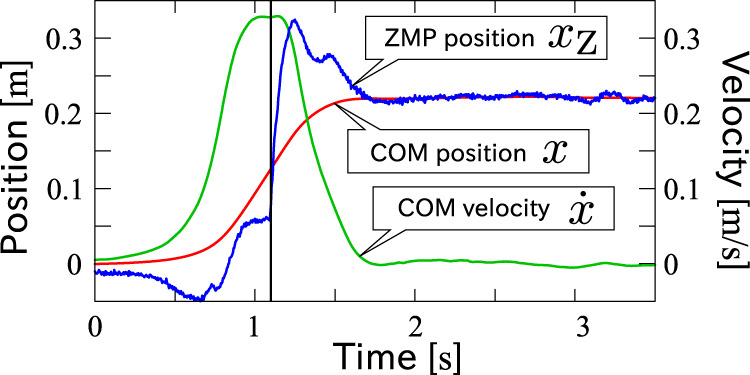
A typical time series of COM position (red line), COM velocity (green line) and ZMP (blue line) of step-and-brake motions. The black vertical line indicates the detected moment of foot-landing.

A typical time series of motion is plotted in Figure 6. The black line around 1.1 s indicates the moment at which the reaction force to the stepping foot exceeded a threshold, and thus, the subject was thought to land her foot on the force plate. The trajectory is separated by the moment into two phases. Let us call the former the stepping phase and the latter the braking phase. While the COM moved smoothly from the initial to the final position over the phase transition, the ZMP presents a distinctive history in each phase. It was initially pulled back in order to accelerate the COM, gradually moved forward, and was finally saturated at the edge of the pivot foot in the stepping phase. In the braking phase, the ZMP went forward and overtook the COM to decelerate it immediately after landing, and brought it smoothly into the referential position. Finally, the COM and the ZMP converged to the same position.

**FIGURE 7:**
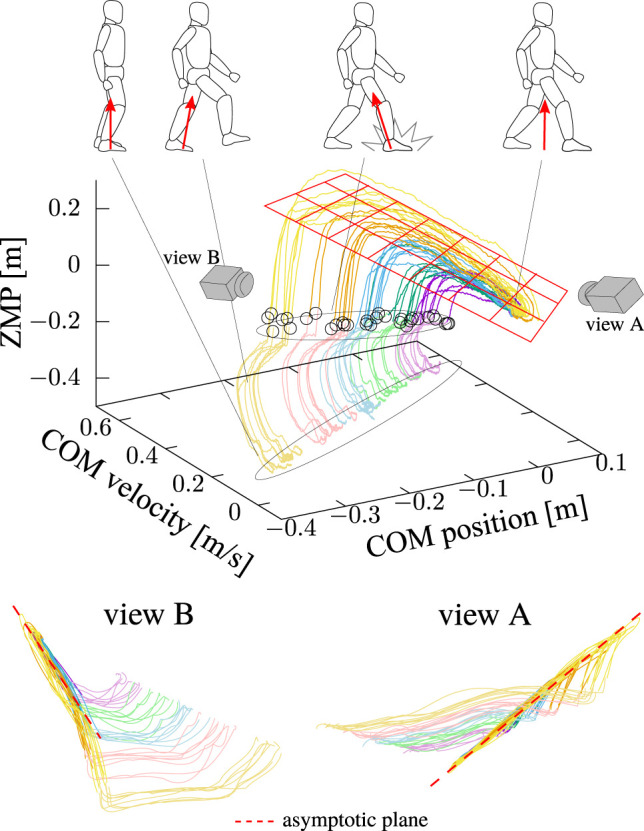
Collected trajectories of step-and-brake motions in *x*- $\dot{x}$ -*x*_Z_ space. The final position of each trajectory is reset to be the original point. Trajectories with the same foot-landing destination are grouped by the same color. Black circles indicate the detected moments of foot-landing on each trajectory. Figures from two different viewpoints implicate the existence of an asymptotic plane (red dashed lines).

Figure 7 shows the measured motion trajectories collected in *x*- $\dot{x}$ -*x*_Z_ space, where the final position of each trajectory is reset to be the original point. Trajectories of motions with the same foot-landing destination are grouped by the same color. The black circles indicate the moments of foot-landing. It is observed that the overall shapes of the trajectories are similar, and also that they asymptotically converge to the same plane in the *x*- $\dot{x}$ -*x*_Z_ space (see view A and B in the figure). This reminds us of the controller Eq. 4, which is the piecewise-linear state feedback, and hence, the trajectories of $\left(x,\dot{x},x_{\text{Z}}\right)$ in that motion are on a plane.

**FIGURE 8:**
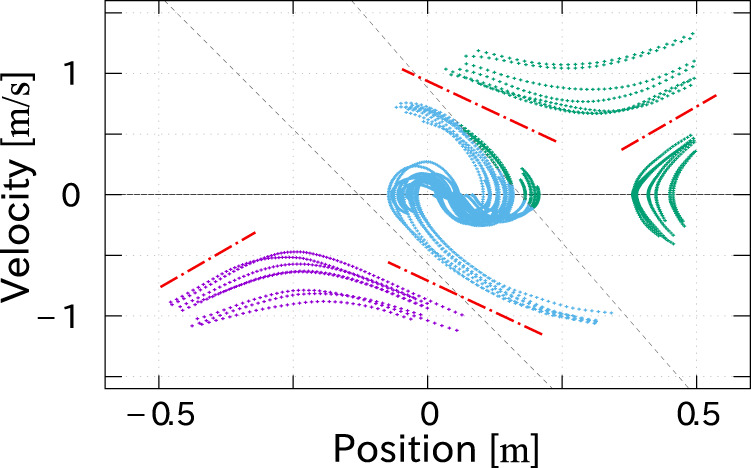
Trajectories of standing motions in *x*- $\dot{x}$ space. The subject took the final posture of the step-and-brake motion with 45 cm stride. The clustering technique proposed in (Sugihara et al., 2022) divided the trajectories into three segments by two black dashed lines. The asymptotic lines (red chained lines) are asymmetric.

**FIGURE 9:**
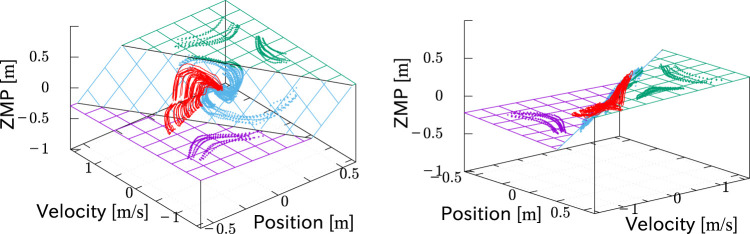
Trajectories of standing motions (cyan, green and purple) in *x*- $\dot{x}$ -*x*_Z_ space from two different viewpoints. Hatched planes in cyan, green and purple represent identified subspaces (S1), (S2) and (S3), respectively. Trajectories of step-and-brake motions (red) are superposed on it. The asymptotic plane of the latter trajectories coincides with the segmented plane of the convergent motions of the standing (hatched in cyan).

In order to investigate the above observation, standing stabilization motions in the sagittal plane were measured in accordance with the same protocol with the authors’ previous work (Sugihara et al., 2022). The subject took the final posture of the step-and-brake motion with 45 cm of the landing point from the initial position. The motion trajectories were obtained by perturbing her in stance as shown in Figure 8. It is confirmed that the behavior is modeled as a piecewise-linear system and the asymptotic lines (red chained lines) are not symmetric as well as the lateral standing measured in the previous work. The clustering technique proposed in (Sugihara et al., 2022) successfully divided the *x*- $\dot{x}$ space into three segments, which are redrawn in the *x*- $\dot{x}$ -*x*_Z_ space with the trajectories of the step-and-brake motions superposed in Figure 9. This figure shows that the asymptotic plane of the latter trajectories coincides with the segmented plane of the convergent motions of the standing (hatched in cyan).

## Mathematical Model of a Step-And-Brake Controller

The result in the previous section obviously showed that the piecewise-linear state feedback Eq. 5 does not fit the observed step-and-brake behavior, though the behavior finally converges to it. Among several candidates of the mathematical model of the controller, the authors focus on a biped robot control proposed by Sugihara et al. (Sugihara and Yamamoto, 2017; Yamamoto and Sugihara, 2020) and adopt it as the primary model because of its minimal property. To be minimal means that it is derived from the minimal conditions to continue the biped motion stably, namely, to confine the desired ZMP in the pivot sole and to guarantee the terminal capturability. It also requires the minimal number of state variables to feedback, which are position and velocity of the COM as well as the COM-ZMP regulator described in Section 2 — the acceleration of the COM is not referred unlike other conventional control schemes (Kajita et al., 2003; Herdt et al., 2010). Accordingly, the controller depends on the minimal number of parameters, which are the locations of the pivot foot, the desired position of the capture point and the motion duration. In spite of the above minimal formulation, it can achieve both the stepping and braking motions in a unified manner.

**FIGURE 10:**
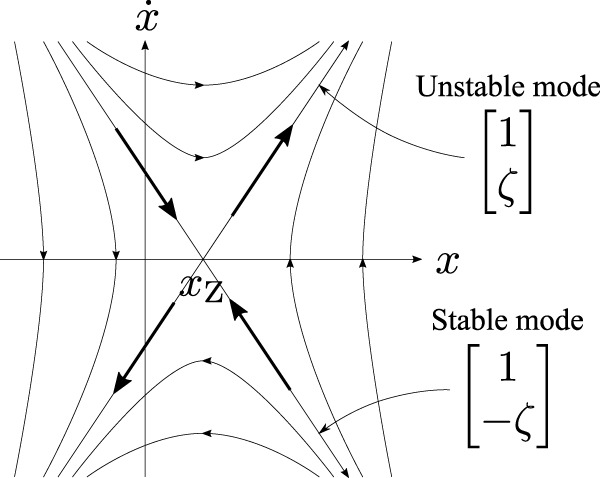
phase portrait of the system Eq. 1 with *x*_Z_ ≡const. It has two eigenvalues *ζ* (unstable) and − *ζ* (stable) with symmetric asymptotic directions ${\left[1\;\;\zeta \right]}^{\text{T}}$ and ${\left[1\;\;-\zeta \right]}^{\text{T}}$ .

In the original work, the COM-ZMP model represented by Eq. 1 was assumed in order to derive the feedback law. This system with *x*_Z_ ≡const. has two eigenvalues *ζ* (unstable) and − *ζ* (stable) with symmetric asymptotic directions ${\left[1\;\;\zeta \right]}^{\text{T}}$ and ${\left[1\;\;-\zeta \right]}^{\text{T}}$ as illustrated in Figure 10. Based on this, the desired ZMP is determined by solving the following minimization problem.

xZd=argminxZ12∫tTxZ−xP2dts. t.Eq.1andxT+x˙Tζ=xCd, (8)

where *T* is the desired terminal time for landing or braking, *t* is the current time, *x*_P_ is the location of the pivot foot, and ^*d*^ *x*_C_ is the desired position of the capture point. The subjected equality condition other than Eq. 1 in the above means that the capturability condition (Pratt et al., 2006; Koolen et al., 2012) at the final time is satisfied. The problem (8) is analytically solved as

xZd=xP+2xCP−e−ζT−txCPd1−e−2ζT−t, (9)

where

$$x_{\text{CP}}\overset{def}{=}x+\frac{\dot{x}}{\zeta }-x_{\text{P}}$$ (10)

xCPd=defxCd−xP. (11)

^*d*^ *x*_Z_ with the above control law asymptotically converges to *x*_P_. If ^*d*^ *x*_C_ = *x*_P_, *x* also stably converges to *x*_P_. Otherwise, *x* is destabilized and diverges toward ^*d*^ *x*_C_. The former and latter lead to the braking and the stepping motions, respectively. Hence, the stepping and braking controls are switched only by unequalizing and equalizing *x*_P_ and ^*d*^ *x*_C_, respectively. In addition, *x*_CP_ also asymptotically converges to ^*d*^ *x*_CP_, which might explain the process in which the trajectories of the COM converges to a plane as shown in Figure 7.

On the other hand, the actual behavior of the human’s COM exhibits direction-dependent modes of falling as portrayed in Figure 8. Hence, we take this into account and employ the following modified equation of motion:

$$\ddot{x}=\zeta_{1}\zeta_{2}\left(x-x_{\text{Z}}\right)+\left(\zeta_{1}-\zeta_{2}\right)\dot{x}.$$ (12)

**FIGURE 11:**
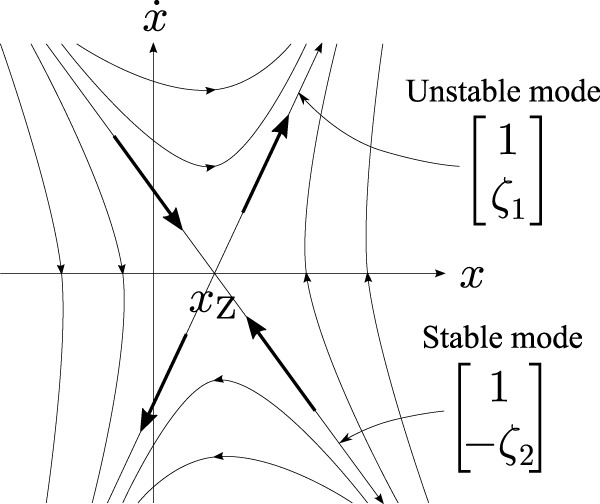
phase portrait of the system Eq. 12 with *x*_Z_ ≡const. It has two eigenvalues *ζ*_1_ (unstable) and − *ζ*_2_ (stable) with asymmetric asymptotic directions ${\left[1\;\;\zeta_{1}\right]}^{\text{T}}$ and ${\left[1\;\;-\zeta_{2}\right]}^{\text{T}}$ .

This system with *x*_Z_ ≡const. has two eigenvalues *ζ*_1_ (unstable) and − *ζ*_2_ (stable) with asymmetric asymptotic directions ${\left[1\;\;\zeta_{1}\right]}^{\text{T}}$ and ${\left[1\;\;-\zeta_{2}\right]}^{\text{T}}$ as illustrated in Figure 11. The corresponding optimization problem is also modified as

xZd=argminxZ12∫tTxZ−xP2dts. t.Eq.12andxT+x˙Tζ2=xCd. (13)

This is also analytically solved as

xZd=xP+2xCP−e−ζ1T−txCPd1−e−2ζ1T−t, (14)

where *x*_CP_ is redefined as

$$x_{\text{CP}}\overset{def}{=}x+\frac{\dot{x}}{\zeta_{2}}-x_{\text{P}}.$$ (15)

## Result of Parameter Identification and Discussion

A system identification from the motion trajectories in Figure 7 was conducted based on Eq. 14 with a modification to take the effect of the inertial torque about the COM into account approximately as

xZd=xP+2xCP−e−ζ1T−txCPd1−e−2ζ1T−t+cL˙, (16)

where *L* is the angular momentum about the COM divided by the vertical ground reaction force and *c* is a constant coefficient. The parameters to be identified in the above equation are *x*_P_, ^*d*^ *x*_C_, *ζ*_1_, *ζ*_2_, *c* and *T*. Since the motion is separated into the stepping and braking phases, we have two corresponding sets of the above parameters. Thus, we distinguish them as *x*_P1_, ^*d*^ *x*_C1_, *ζ*_11_, *ζ*_21_, *c*_1_ and *T*_1_ for the stepping phase, and as *x*_P2_, ^*d*^ *x*_C2_, *ζ*_12_, *ζ*_22_, *c*_2_ and *T*_2_ for the braking phase, respectively. In order to simpify the problem to be solved, *T*_1_ and *T*_2_ were determined as the time when the measured reaction force and the measured acceleration of the COM exceeded the corresponding thresholds, respectively, in advance. Then, the remaining parameters were identified separately in each phase by solving the following least square minimization problem:

∑k=0N*12xP*+2xCP*k−e−ζ1T*−kΔtxCP*d1−e−2ζ1T*−kΔt+c*L˙nk−xZk2→min.s.t.xCP*N*=xN*+x˙vN*ζ2*−xP*, (17)

where * = 1 or 2, *k* is the discretized time index,

$${x_{\text{CP}}}_{*}\left[k\right]=x\left[k\right]+\frac{\dot{x}v\left[k\right]}{\zeta_{2*}}-x_{\text{P}*}$$ (18)

xCP*d=xC*d−xP*, (19)

*Δt* is the sampling interval, and N_*_ = T_*_/*Δt*. [*k*] denotes that the associated quantities were sampled at *t* = *kΔt*. The above problem was solved by the steepest descent method, where the gradient was numerically estimated by finite difference.

**FIGURE 12:**
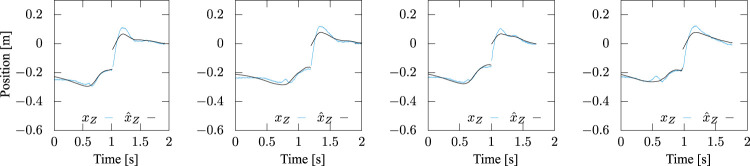
Measured and identified ZMP trajectories based on Eq. 14, in which the inertial torque about COM was ignored. Step stride was controlled to be 45 cm. While the latter reproduced the former well in the stepping phase, substantial errors are observed after landing.

**FIGURE 13:**
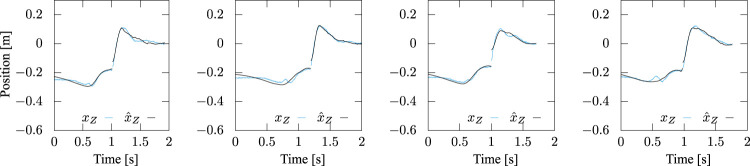
Measured and identified ZMP trajectories based on Eq. 16, in which the inertial torque about COM is taken into account. Step stride was controlled to be 45 cm. The latter reproduced the former well in both the stepping and braking phases.

**FIGURE 14:**
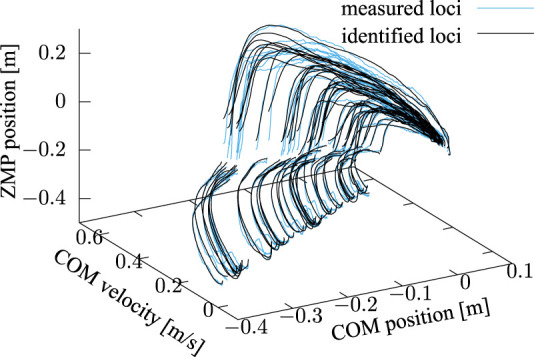
Trajectories of *x*- $\dot{x}$ -*x*_Z_ measured (cyan) and reproduced (black) from identified parameters based on Eq. 16. The latter qualitatively show good fits to the former due to the additional term of inertial torque about COM.

Figures 12, 13 present the trajectories of the ZMP of the motions with a stride of 45 cm based on Eqs 14, 16, respectively. The cyan lines in the figures are the measured trajectories, while the black lines were reproduced from the identified parameters. The figures show that Eq. 14 captures characteristics of the time profile of the ZMP and the identification accuracy in the braking phase after the foot-landing was improved due to the additional term in Eq. 16, while it is not much in the stepping phase. All the trajectories of *x*- $\dot{x}$ -*x*_Z_ measured and reproduced from the identified parameters based on Eq. 16 are plotted in Figure 14 in the same colors.

**FIGURE 15:**
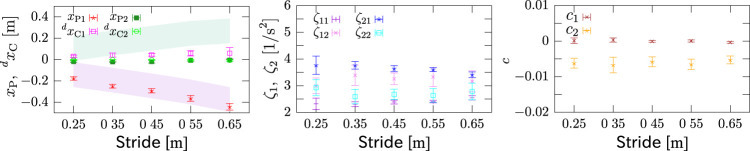
Identified parameters. Left: positions of pivot point and landing destination. Final position of COM is reset to be the original point. Magenta and green areas represent soles of pivot and landing feet, respectively. *x*_P1_ stays in pivot sole, and *x*_P2_, ^*d*^ *x*_C1_ and ^*d*^ *x*_C2_ are around 0. Middle: eigenvalues in each phase. The unstable eigenvalue *ζ*_1*_ increased after landing, i.e., *ζ*_11_ < *ζ*_12_, while the stable eigenvalue *ζ*_2*_ decreased, i.e., *ζ*_21_ > *ζ*_22_. Right: coefficient of inertial torque about COM. *c*_1_ ≃ 0 but *c*_2_ ≠ 0. All ζ_*_s and c_*_s are almost constant irrespective of step width, which supports the model.

Figure 15 shows the result of the identification of each parameter. In the left figure, the final position of the COM is reset to be the original point. The magenta and green areas show soles of the pivot and landing feet, respectively. The result is consistent with the predictable values in which *x*_P1_ would stay within the pivot sole and *x*_P2_, ^*d*^ *x*_C1_ and ^*d*^ *x*_C2_ would be around 0. The middle figure shows that *ζ*_1_ and *ζ*_2_ in each phase were also fairly identified with small variances. An issue to be noted here is that the unstable eigenvalue increased after landing, i.e., *ζ*_11_ < *ζ*_12_, while the stable eigenvalue decreased, i.e., *ζ*_21_ > *ζ*_22_, and the magnitude relationship between the stable and unstable eigenvalues is inverted after landing, i.e., *ζ*_11_ < *ζ*_21_ and *ζ*_12_ > *ζ*_22_. The right figure reads that the effect of the inertial torque about the COM is almost negligible in the stepping phase, i.e., *c*_1_ ≃ 0, while it is not in the braking phase. The reasons of these are currently unclear and should be discussed in the future. For all ζ_*_s and c_*_s, they are almost constant irrespective of the stride, which also supports the model.

**FIGURE 16:**
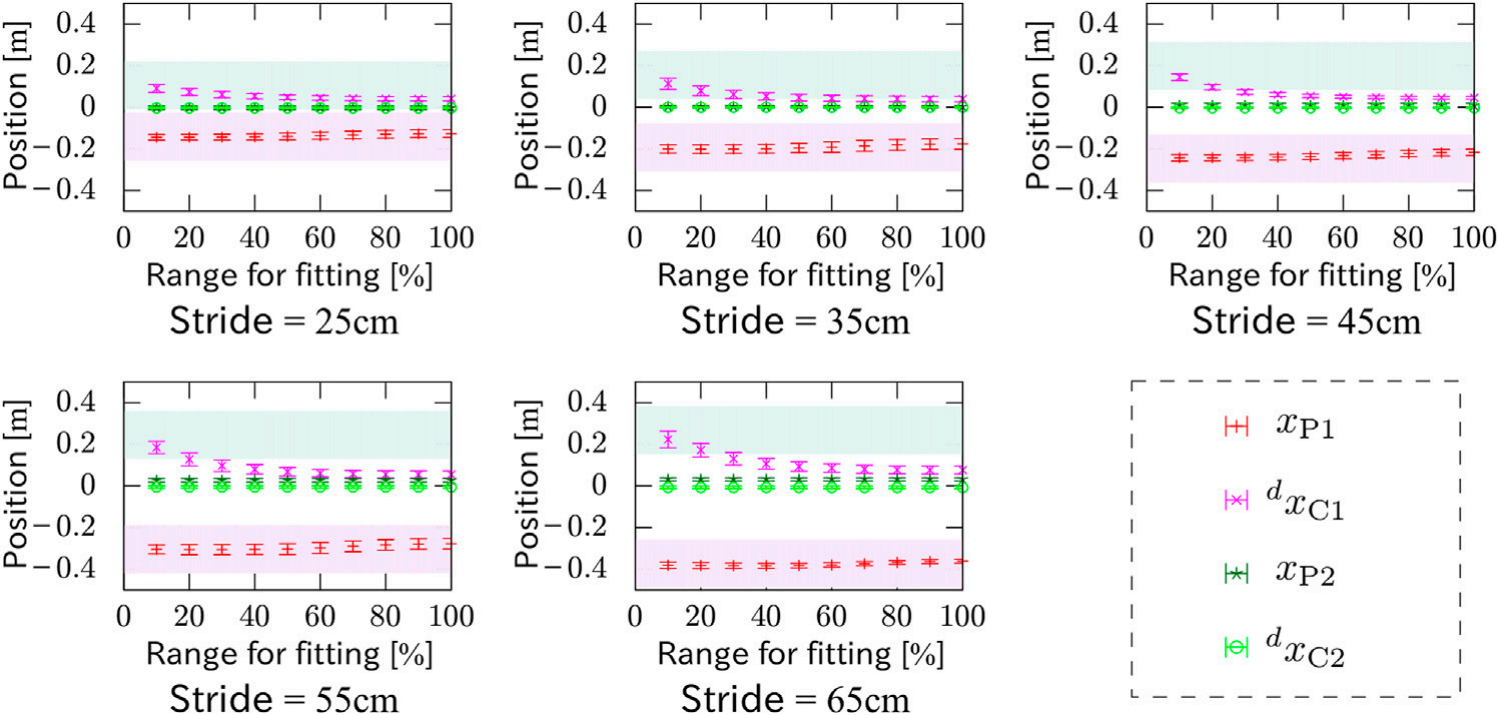
*x*_P1_, *x*_P2_, ^*d*^ *x*_C1_ and ^*d*^ *x*_C2_ estimated from partial observations in time of experiments with different strides. The abscissa means the percentage of the observation with respect to the whole motion data.

**FIGURE 17:**
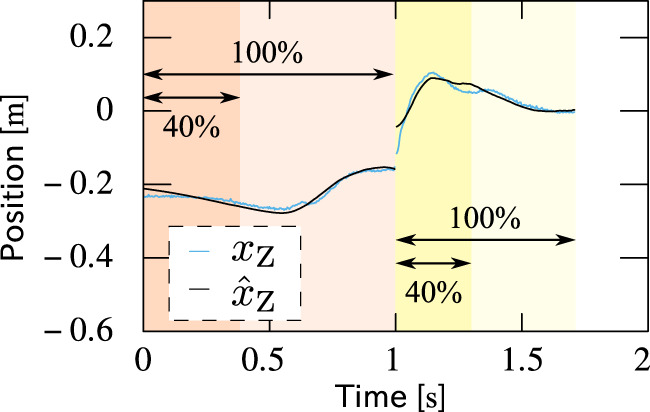
Comparison between measured and reproduced trajectories of the ZMP. The latter was reproduced only from first 40% of the observed motion data in each phase but fits the former.

More interestingly, it was verified that the parameters can be reasonably estimated only from partial observation of the motion data in time. Figure 16 shows *x*_P1_, *x*_P2_, ^*d*^ *x*_C1_ and ^*d*^ *x*_C2_ of experiments with different strides, where the abscissa means the percentage of the observation with respect to the whole data. The trajectory of the ZMP of the measured motion with a stride of 45 cm reproduced only from first 40% of the observed motion data in each phase is plotted in Figure 17, which fits the measured trajectory. Remember that *x*_P_ ≠^*d*^ *x*_C_ and *x*_P_ = ^*d*^ *x*_C_ correspond to the stepping and braking motions, respectively. This implies that the intention, *i.e.*, to step or to brake, of the measured subject can be guessed before completing even a half of the action.

## Conclusion and Future Directions

A mathematical model of a step-and-brake control of a human subject was identified in a form of Eq. 16. The plausibility of the model at least with respect to the measured subject was shown as it reproduced trajectories of the COM and the ZMP in the overall motion process with a fair accuracy, which is a clear contribution of this work over the previous researches that focused on the capturability condition (Hof, 2008; Aftab et al., 2012). Note that we do neither mean to generalize the above discussion with only one subject nor immediately conclude that the identified model is best-suited for modeling of the behavior, but we think it could suggest the start point of discussions. Even though behaviors of only one subject were investigated in this work, highly resemblant output of the identified model to the measured data seems to us more than coincidental. It should be improved to be general through case studies and statistics of more subjects.

It was also shown that the control parameters could be estimated only from the first half movement before completing the action, with which the intention to step or to brake of the subject could be guessed. Thus, the authors think that the model is utilized in designing wearable assistive devices for human walking to recover balances and systems to evaluate humans’ balancing abilities on the fly. It is also an advantage of the proposed method for this purpose that it does not require a detailed body model of a human subject. We expect that the proposed controller model and protocol to identify it for individuals will benefit improvement of the quality of life of elderly and disabled people by detecting irregular behaviors of users only from their anticipatory movements and suggesting appropriate supportive actions of such devices.

On the other hand, there are still some technical issues to be considered for practical applications as well as how to measure the trajectory of the COM in real-time. It has to predict the remaining time to land and brake in advance in order to identify the parameters. Also, the effect of the inertial torque about the COM should be compensated in order to improve the accuracy. It is not trivial how to resolve the above problems and should be overcome in the future.

## Data Availability

The datasets presented in this article are not readily available because it includes personal information. Requests to access the datasets should be directed to zhidao@ieee.org.
